# Identification of Halophilic and Halotolerant Bacteria from the Root Soil of the Halophyte *Sesuvium verrucosum* Raf

**DOI:** 10.3390/plants11233355

**Published:** 2022-12-02

**Authors:** Javier Pérez-Inocencio, Gabriel Iturriaga, Cesar L. Aguirre-Mancilla, Juan Gabriel Ramírez-Pimentel, María Soledad Vásquez-Murrieta, Dioselina Álvarez-Bernal

**Affiliations:** 1 Tecnológico Nacional de México Campus Roque, Celaya 38525, Mexico; 2 Escuela Nacional de Ciencias Biológicas, Instituto Politécnico Nacional, Mexico City 07738, Mexico; 3 Instituto Politécnico Nacional, Centro Interdisciplinario de Investigación para el Desarrollo Integral Regional Unidad Michoacán (CIIDIR-Michoacán), Jiquilpan 59510, Mexico

**Keywords:** plant growth promoter, salinity, *Solanum lycopersicum*, sodium chloride, germination

## Abstract

Soil salinity is a condition that limits crop growth and productivity, and soil-dwelling bacteria from halophytic plant roots may be a viable strategy to cope with low productivity due to salt stress. Halophilic and halotolerant bacteria of the root soil of *Sesuvium verrucosum* were analyzed in this study as there is little evidence regarding its associated microbiology. Soil was sampled from the roots of *Sesuvium verrucosum* to obtain the cultivable bacteria. Their morphological characteristics were identified and they were molecularly identified by the 16S sequence. The growth capacity of the bacteria was determined at different levels of pH and salinity, and several growth promotion characteristics were identified, such as phosphorus solubilization, indole acetic acid production by the tryptophan-dependent (AIAt) and tryptophan-independent (IAA) pathways, ammonium production from organic sources, solubilization of carbonates, and zinc and sodium capture capacity. In addition, the bacteria that presented the best characteristics for germination variables of *Solanum lycopersicum* were evaluated. A total of 20 bacteria from root soil of *Sesuvium verrucosum* Raf. belonging to the phyla Proteobacteria (50%), Firmicutes (45%) and Actinobacteria (5%) were identified, with each one having different morphological characteristics. Among the bacterial isolates, 45% had the ability to resist different levels of salinity and pH, ranging from 0 to 20% of NaCl, and pH between 5 and 11. Moreover, these bacteria had the capacity to solubilize carbonates, phosphorus and zinc, capture sodium, produce ammonium from organic substrates and IAA (indole acetic acid), and promote enzymatic activity of amylases, proteases, lipases and cellulases. The bacteria evaluated on the germination of *Solanum lycopersicum* had an influence on germination at different salinity levels, with greater influence at 100 mM NaCl. This demonstrated that halophilic bacteria belonging to the rhizosphere of *Sesuvium verrucosum* have the ability to promote growth in extreme salinity conditions, making them candidates for the recovery of productivity in saline soils.

## 1. Introduction

The increase in agricultural production in order to meet the world’s growing demand for food has brought different types of problems, such as the chemical and physical degradation of the soil, thereby reducing its productive potential [1,2]. Worldwide, salinity is the main threat to agricultural production [2,3,4], affecting between 7 and 10% of the continental territory [5].

Plants that inhabit saline soils adapt to salinity conditions without compromising their growth, incorporating and accumulating large amounts of salts. These plants are known as halophytes [6]. *Sesuvium verrucosum* Raf. is a perennial halophytic plant of the Aizoaceae family that has a high resistance to salinity [7] and can even modify chemical characteristics of the soil such as pH and electrical conductivity to withstand saline stress [8]. Its tolerance is attributed to different mechanisms such as biochemical, morphological and structural changes [9]; however, its adaptive capacity is also attributable to the association it forms with soil bacteria that have the ability to improve plant growth [10,11]. These bacteria are known as plant growth promoter rhizobacteria (PGPR) [12].

The use of halophilic microorganisms is an alternative for the bioremediation of saline soils due to their ability to absorb salts, as well as their capacity to promote plant growth that can influence metabolic activities, nitrogen fixation, enzymatic activity of 1-aminocyclopropane-1-carboxylate (ACC) deaminase, siderophore synthesis, phytohormone production, hydrolase enzyme activity [13,14,15] and activation of antioxidants, exopolysaccharides and genes responsible for the attenuation of salt stress [16,17]. These properties allow halophilic bacteria to promote plant growth and resistance to abiotic stress [3,18].

Several studies have isolated bacteria from the rhizosphere of halophytic plants and have shown their plant growth promoting characteristics, such as IAA (indole acetic acid) and siderophore production, phosphate solubilization [19,20] and enzymatic activity [21]. Kearl et al. [22], isolated bacteria from the rhizosphere of the halophytes *Salicornia rubra*, *Sarcocornia utahensis* and *Allenrolfea occidentalis.* Other studies have used *Capparis decidua* [23], *Crithmum* sp. [14], *Atriplex* sp. [14,15], *Salsola stocksii* [15], Jerusalem artichoke [24], *Aster tripolium* [25] and *Sauceda* sp. [26], to name a few. Moreover, some studies have used plant growth promoting microorganisms from the rhizosphere of halophytic plants in order to attenuate the effect of salt stress on crops of economic importance, such as *Beta vulgaris* L. [27], *Medicago sativa* [22], *Salicornia* sp. [28], *Triticum aestivum* L. [29], *Solanum lycopersicum* [14], *Arabidopsis thaliana*, *Cucumis sativus* [30], and *Zea mays* L. [31], among others. Nonetheless, there is scarce information about bacteria associated with the rhizosphere of *S. verrucosum* and their ability to provide the plant with some characteristics that promote its growth. Among the few works that describe bacteria associated with *S. verrucosum*, we found research conducted by El-Awady et al. [10], who reported the isolation and characterization of endophytic bacteria from the rhizosphere of this halophyte, identifying their phosphorus solubilization capacity, IAA production and nitrogen fixation. *Sesuvium verrucosum* is a species native to the Municipality of Villamar, Michoacán, Mexico and it has a high capacity for resistance to saline stress [8]. The rhizospheric microbiology of this species and role the bacteria could play in its resistance to saline stress are little studied. In addition, there are no reported works on the enzymatic activity of hydrolases, sodium capture capacity, zinc solubilization, carbonate solubilization or ammonium production from an organic source by *Sesuvium verrucosum* root soil bacteria, hence the relevance of this research.

Considering the aforementioned, the objective of this work was to identify microorganisms from the root soil of *Sesuvium verrucosum* growing in saline soils and their capacity to produce compounds that promote plant growth and production of hydrolase enzymes, in order to be able to use them in the future to attenuate salt stress in crops of agricultural interest.

## 2. Results

### 2.1. Soil Characterization

The salinity characterization of the root soil of *S. verrucosum* was carried out using the saturated paste extract method (Table 1). The soil was classified as highly saline based on the electrical conductivity and had a moderately alkaline value of pH. The concentration of Ca^2+^ (24.6 meq/L) was 1.8 times more than Mg^2+^ and 2.4 times that of K^+^. The Na^+^ concentration (206 meq/L) exceeded by more than 4.29 times the concentrations of Ca^2+^, Mg^2+^ and K^+^**.** The ratio of Na^+^ to other cations is shown by the exchangeable sodium percentage (40.6) and the sodium adsorption ratio (47.3), both being high values. On the other hand, anion concentrations were high for SO_4_^−^ (111 meq/L) and Cl^−^ (138 meq/L), but low for HCO_3_^−^ (3.87 meq/L) and CO_3_^−^ (2.71 meq/L). Hence, this soil was considered saline-sodic.

### 2.2. Morphology of the Isolates

A total of 20 bacteria were isolated from the root soil of *S. verrucosum*. The bacterial isolates presented different morphological characteristics: 60% had an irregular form, 25% circular, 10% punctiform and only 5% oval. Regarding elevation, 65% were flat, 25% raised and 10% convex. An entire margin prevailed with 65%, followed by 25% undulate and only 10% lobate. The predominant surface of the colonies was smooth (60%), and the rest had a rough surface. The color that predominated in the bacterial colonies was cream with 70%, then yellow (20%) and orange (10%). The creamy consistency (65%) prevailed compared with the dry consistency (Table S1).

From the 20 bacterial strains, 40% were Gram-positive and 60% Gram-negative. With regard to their shape and arrangement, 75% of the isolates presented a bacillus shape and the rest a coccus shape, while 50% had a bacillus arrangement, 20% staphylococcus, 15% diplobacillus, 10% palisade and only 5% streptobacillus (Table S1).

### 2.3. Molecular Identification

**Figure 1:**
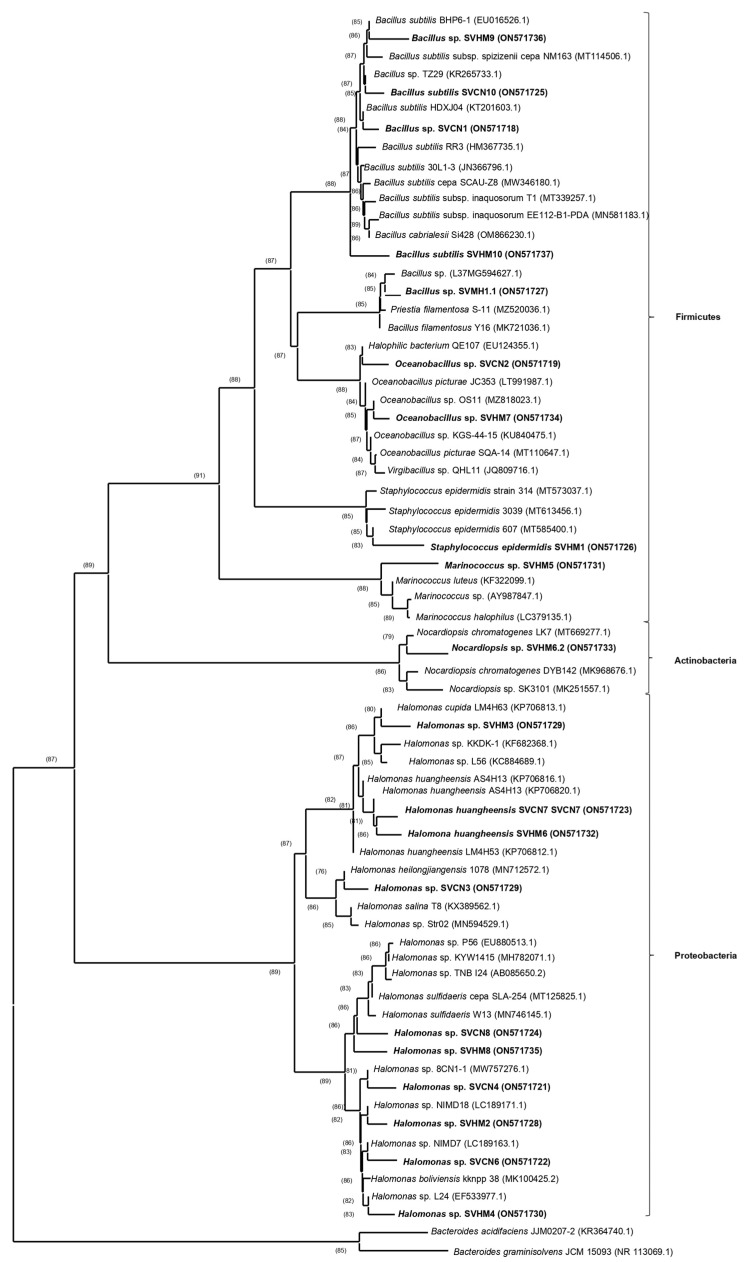
Phylogenetic cladogram of bacterial isolates from the root soil of *S. verrucosum*. The sequences used to construct the phylogenetic cladogram were obtained from selected bacterial databases based on BLAST results of 16S rRNA sequences identified by their GenBank accession number. The numbers on branches indicate bootstrap support (>50%) with at least 1000 replicas. The figures in parentheses indicate the percentage of coverage of the site. *Bacteroides acidifaciens* JJM0207-2 and *Bacteroides graminisolvens* JCM 15,093 are included as an outgroup.

The 16S rRNA gene sequences of the 20 bacteria isolated from the root soil of *S. verrucosum* were deposited in the GenBank database under accession numbers ON571718 to ON571737 (Table S2). The isolates belonged to the phyla Proteobacteria (50%), Firmicutes (45%) and Actinobacteria (5%), and were identified in six genera: *Halomonas* (50%), *Bacillus* (25%), *Oceanobacillus* (10%), *Marinococcus* (5%), *Staphylococcus* (5%) and *Nocardiopsis* (5%), which meant a relative abundance of the families *Halomonadaceae* (50%), *Bacillaceae* (40%), *Nocardiopsaceae* (5%) and *Staphylococcaceae* (5%). Table S2 shows that the genus *Halomonas* prevailed among the isolates, with the following isolates being ≥99.07% similar to the reference strains: SVCN3, SVCN4, SVCN6, SVCN7, SVCN8, SVHM2, SVHM3, SVHM4, SVHM6 and SVHM8. The isolates SVCN1, SVCN10, SVHM1.1, SVHM9 and SVHM10 were ≥97.72% similar to the genus *Bacillus*; whereas SVCN2 and SVHM7 were ≥99.79% similar to the genus *Oceanobacillus*. Lastly, the isolates SVHM1, SVHM5 and SVHM6.2 presented 99.85% similarity to the *Staphylococcus* genus, 99.44% to the *Marinococcus* genus and 98.94% to the *Nocardiopsis* genus, respectively. In addition, based on the results obtained from the alignment of the base pairs of each isolate and the comparison of sequences using the NCBI databases, all the bacterial sequences were aligned and analyzed to obtain a phylogenetic cladogram (Figure 1).

### 2.4. Evaluation of Tolerance to Salinity, pH and Sodium Capture Capacity

The behavior of the bacterial isolates at different pH levels demonstrates the bacterial variability in the root soil of *S. verrucosum*. Most bacterial strains grew optimally at a pH between 6 and 11. Only the isolates *Bacillus* sp. SVHM9, *Bacillus subtilis* SVHM10, *Oceanobacillus* sp. SVCN2, *Marinococcus* sp. SVHM5, *Halomonas* sp. SVCN6, *Halomonas* sp. SVCN8, *Halomonas* sp. SVHM3 and *Halomonas* sp. SVHM4 showed growth capacity at a pH of 5, whereas the isolate *Nocardiopsis* sp. SVHM6.2 could only grow at a pH of 7. At a pH higher than 10, most of the isolates showed growth capacity, except for *Bacillus* sp. SVCN1, *Staphylococcus epidermidis* SVHM1 and *Bacillus* sp. SVHM9, which grew at a maximum pH of 10, while the isolate *Halomonas* sp. SVHM6 could grow at a maximum pH of 8 (Table 2).

Regarding the growth at different salinity concentrations (Table 3), most of the isolates grew at NaCl concentrations ranging from 0 to 20% (equivalent to 0–3.42 mol/L NaCl), showing significant growth at concentrations between 2.5 and 10% NaCl. The bacterial strains *Oceanobacillus* sp. SVCN2, *Halomonas* sp. SVCN3 and *Nocardiopsis* sp. SVHM6.2 did not grow at 0% NaCl. This last strain only showed growth capacity from a concentration of 5% NaCl. While at concentration levels of 15, 20 and 25% only some strains could grow, the strain *Marinococcus* sp. SVHM5 stood out for growing at all salinity levels.

The sodium capture capacity of some of the root soil isolates of *S. verrucosum* has been evidenced in this work. It is worth mentioning that this is the first work to report that there are bacteria associated with the rhizosphere of this halophyte that can capture sodium. The isolates that presented sodium capture capacity were *Bacillus* sp. SVCN1, *Bacillus* sp. SVHM1.1, *Bacillus* sp. SVHM9, *Bacillus subtilis* SVCN10, *Bacillus subtilis* SVHM10, *Oceanobacillus* sp. SVCN2, *Oceanobacillus* sp. SVHM7, *Staphylococcus epidermidis* SVHM1, *Marinococcus* sp. SVHM5, *Nocardiopsis* sp. SVHM6.2, *Halomonas* sp. SVCN3, *Halomonas* sp. SVCN8, *Halomonas* sp. SVHM3, *Halomonas* sp. SVHM8, *Halomonas huangheensis* SVCN7 and *Halomonas huangheensis* SVHM6, ranging from 11 mEq (*Bacillus subtilis* SVHM10) to 38 mEq with higher production (*Halomonas huangheensis* SVHM6) after 24 h of incubation (Table S3). The the other isolates showed no reduction in the initial concentration of sodium in the culture medium.

### 2.5. Production of metabolites with Characteristics of Growth Promotion and Enzymatic Activity of the Isolates

All the bacterial isolates were tested for plant growth promoting capacity and they presented at least one growth promotion characteristic. It should be noted that isolates *Bacillus* sp. SVHM1.1, *Halomonas* sp. SVCN3, *Halomonas* sp. SVCN4, *Halomonas* sp. SVCN6, *Halomonas* sp. SVHM2 and *Halomonas* sp. SVHM8 presented five or more of the evaluated growth promotion characteristics (Table S3).

With regard to phosphate solubilization capacity, the isolates presented differences from 0 mg/mL to 81 mg/mL. The isolate *Halomonas* sp. SVCN8 did not present any solubilization capacity, whereas *Bacillus* sp. SVHM1.1 showed a solubilization capacity of 81 ± 4.4 mg/mL, being 80% higher compared with the lowest capacity isolate. A total of eleven bacterial isolates presented IAA production capacity by the tryptophan-independent pathway with values ranging from 4.0 ± 1.8 to 9.9 ± 1.5 μg/mL, with *Halomonas* sp. SVCN3 being the isolate with the highest production capacity. Concerning the production of IAA by the tryptophan-dependent pathway, 10 bacterial strains presented this capacity with values ranging from 4.9 ± 0.4 to 20.6 ± 3.8 μg/mL. Once more the isolate *Halomonas* sp. SVHM8 is highlighted with the highest production capacity. In addition to being specific for IAA, it has also been shown that other intermediate IAA compounds are reactive to the Salkowski colorimetric technique, such as idolpyruvic acid (IPA) and idolacetamide acid (IMA) [32]. These two routes are the main ones in the synthesis of AIA [33]. Most of the isolates showed the ability to produce ammonium (NH_4_^+^) from an organic source (from 0 mg/mL to 21.5 ± 1.0 mg/mL); however, the production capacity varied between the strains, with *Bacillus* sp. SVCN1 having the highest production capacity (21.5 ± 1.0 mg/mL), whereas *Oceanobacillus* sp. SVCN2, *Oceanobacillus* sp. SVHM7, *Halomonas* sp. SVHM3, *Marinococcus* sp. SVHM5 and *Nocardiopsis* sp. SVHM6.2, did not present ammonium production capacity. On the other hand, 10 isolates had the capacity to solubilize carbonates. *Bacillus subtilis* SVCN10 had the greatest activity coefficient (8.5 ± 2.1), followed by *Halomonas* sp. SVHM8 with a coefficient of 6.7 ± 0.4, whereas *Halomonas* sp. SVCN6 showed a lower capacity for carbonate solubilization with an activity coefficient of 1.5 ± 0.4. With respect to zinc solubilization capacity, only the isolate *Bacillus* sp. SVHM1.1 presented this characteristic, having an activity coefficient equivalent to 0.7 ± 0.1 (Table S3).

Regarding enzymatic production, some isolates had one or more capacities to produce hydrolase enzymes (lipases, proteases, amylases or cellulases). The isolates *Oceanobacillus* sp. SVCN2, *Marinococcus* sp. SVHM5, *Halomonas* sp. SVCN8, *Halomonas* sp. SVHM2, *Halomonas* sp. SVHM4 and *Halomonas huangheensis* SVCN7 presented capacity for lipase activity. Only the isolates *Halomonas* sp. SVCN4 and *Halomonas* sp. SVCN6 showed the capacity for protease activity. However, the isolates *Bacillus* sp. SVCN1, *Bacillus* sp. SVHM9, *Bacillus subtilis* SVCN10, *Bacillus subtilis* SVHM10 and *Halomonas huangheensis* SVCN7 had the capacity for amylase activity, while the isolates *Bacillus* sp. SVCN1, *Bacillus* sp. SVHM9, *Bacillus subtilis* SVCN10, *Bacillus subtilis* SVHM10, and *Halomonas* sp. SVCN3 presented capacity for cellulase activity (Table S3).

**Figure 2:**
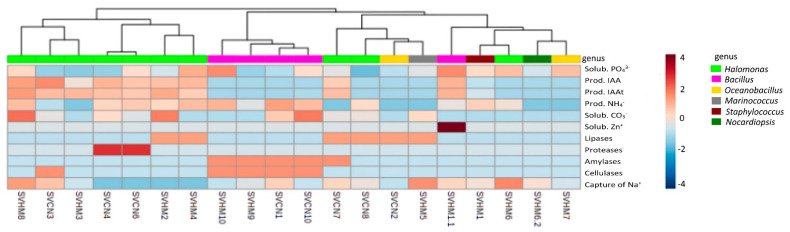
Heat map of the characteristics of plant growth promotion and enzymatic activity of bacterial isolates associated with the root soil of *S. verrucosum*. Phosphate solubilization (Solub. PO_4_^3−^); Tryptophan-independent IAA production (Prod. IAA); Tryptophan-dependent IAA production (Prod. IAAt); Ammonium production from organic sources (Prod. NH_4_^+^); Carbonate solubilization (Solub. CO_3_^−^); Zinc solubilization (Solub. Zn^+^); Sodium capture capacity (Capture of Na^+^). The values of the scale range from −4 to 4; values close to −4 indicate no capacity and values close to 4 indicate high capacity for some of the corresponding characteristics.

Based on the characteristics of growth promotion and enzymatic production capacity of the 20 isolated bacterial strains associated with the root soil of *S. verrucosum*, a heat map was created to determine the similarity between the isolates. As shown in Figure 2, the bacteria were grouped into two large groups: those with characteristics of greater growth promotion and enzymatic activity and those that presented less activity in both areas. Those that presented the greatest number of growth-promoting characteristics and hydrolase enzyme production activity were the isolates SVHM8, SVCN3, SVHM3, SVCN4, SVCN6, SVHM2 and SVHM4, all of which belonged to the same genus (*Halomonas*). A second group with less favorable characteristics was divided into two subgroups: those with some enzymatic activity and those with some plant growth promoting characteristics. Bacteria of the genus *Bacillus* showed some enzymatic activity related to growth promotion. The other subgroup of bacteria that presented some or no growth promoting characteristics or enzymatic activity consisted of the bacterial genera *Oceanobacillus*, *Marinococcus*, *Staphylococcus* and *Nocardiopsis*.

### 2.6. Promotion of Plant Growth in Germination of Solanum lycopersicum under Saline Stress

**Figure 3:**
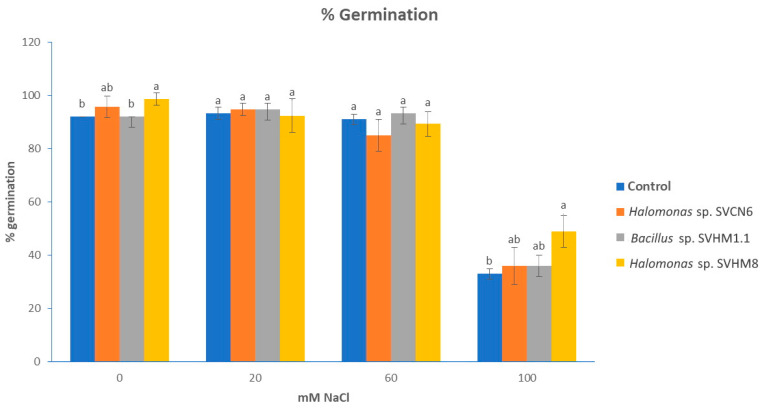
Effect of halophilic bacteria with growth promoting characteristics on *Solanum lycopersicum* germination. The y-axis shows the percentage of germination, and the x-axis indicates the salinity levels in mM (millimoles) of NaCl. Statistical analysis was performed comparing treatments at each salinity level. Bars indicate standard error; different letters at each salinity level indicate significant differences between treatments, Tukey’s test (*p* ≤ 0.05).

The germination of *Solanum lycopersicum* seeds was evaluated with the isolates (*Halomonas* sp. SVCN6, *Bacillus* sp. SVHM1.1 and *Halomonas* sp. SVHM8) that presented better plant growth promotion characteristics at different salinity levels (0.20, 60 and 100 mM NaCl) (Table S3). Figure 3 shows the results of the germination of *S. lycopersicum*. It can be observed that the higher the concentration of salinity, the greater the negative effect on the germination percentage. For the concentration of 0 mM salinity, there are significant differences between treatments (*p* ≤ 0.05). *Halomonas* sp. SVHM8 showed a higher percentage of germination (98%) compared with the control treatment (92%).

For concentrations of 20 and 60 mM of salinity, there are no significant differences between the inoculated treatments with respect to the control; on the other hand, there is a smaller effect of salinity with *Bacillus* sp. SVHM1.1 at the concentration of 60 mM compared with the same treatment at 0 mM, indicating the ability of the bacteria to resist the effect of stress on the seed.

At a concentration of 100 mM NaCl, the germination percentage is drastically reduced in all treatments; however, this effect is reduced in the *Halomonas* sp. SVHM8 treatment which had a germination percentage of 49% and was statistically significant (*p* ≤ 0.05) compared with the control.

## 3. Materials and Methods

### 3.1. Site Description, Sampling and Soil Characterization

The sampling site—known as “Los Negritos”—is located in the municipality of Villamar, Michoacán, Mexico at an altitude of 1540 m.a.s.l., with coordinates 20°03′54.6″ N and 102°36′52″ W. It has a temperate climate, hot summers, an average annual temperature of 21.2 °C and rainfall from June to September with an annual average of 723 mm. The area is characterized by its clay soils rich in natural salts and a great variety of halophytic species, such as *Trianthema portulacastrum* L., *Sesuvium verrucosum* Raf., *Flaveria trinervia* (Spreng.), *Heliotropium curassavicum* L., *Chenopodium murale* L., *Chloris gayana* Kunth, *Diplachne fusca*, *Distichlis spicata* L., *Rumex mexicanus* Meisn. and *Bacopa monnieri* Wettst [34].

Twenty-five random samples of the root soil of *Sesuvium verrucosum* (approximately 100 g) were taken, dried at room temperature, then ground and sieved through a 2-mm mesh. Following the method described by Richards [35], the pH and electrical conductivity were determined from a saturated soil paste extract using a HANNA benchtop meter (HI5522-01). The concentrations of Ca_2_^+^, Mg_2_^+^, K^+^ and Na^+^ cations were measured using the atomic absorption spectrometer GBC SensAA. The concentrations of soluble anions Cl^−^, CO_3_^−^, HCO_3_^−^ and SO_4_^−^ were determined by volumetry. Lastly, the sodium adsorption ratio (SAR) and the exchangeable sodium percentage (ESP) were calculated using the following equations [35]:

$$\mathrm{SAR}\;=\frac{{\mathrm{Na}}^{+}}{\frac{\sqrt{C_{a}^{2+}+M_{g}^{2+}}}{2}}$$

$$\mathrm{ESP}\;=\frac{100\left(-0.0126+0.01475\mathrm{SAR}\right)}{1+\left(-0.0126+0.01475\mathrm{SAR}\right)}$$

where Na^+^, Ca^2+^ and Mg^2+^ represent the corresponding concentration in meq/L.

### 3.2. Isolation of Bacteria

Subsamples of 10 g were taken from the soil samples and diluted in 90 mL of 1.5% saline solution for 30 min at 150 rpm. Subsequently, 1 mL was transferred to plates with nutrient agar medium (meat extract 1 g/L; yeast extract 2 g/L; peptone 5 g/L; agar 15 g/L) [36] modified with 10% NaCl. After seven days at 28 °C, bacteria were selected based on morphological differences and purified by cross-streaking.

The obtained purified bacteria were cultured in 5 mL of nutrient broth (meat extract 1 g/L; yeast extract 2 g/L; peptone 5 g/L; NaCl 10 g/L) [34], modified with 10% NaCl. The cultures were incubated at 28 °C for 24–48 h. Afterward, 1 mL of the culture was supplemented with 1 mL of 30% of glycerol (*w/v*) and stored at −70 °C.

### 3.3. Bacterial Morphology

The morphology of the purified strains was characterized according to their size, form, elevation, margin, surface texture, color and consistency. The microscopic morphology of the strains was determined by a Gram stain test modified by Dussault for halophilic microorganisms [37], and their shape and microscopic arrangement were observed.

### 3.4. Molecular Identification

The identification of the isolates was carried out by extracting genomic DNA using the modified protocol of Hoffman and Winston [38]. The amplification of the 16S rRNA gene was carried out using the primers 27F and 1492R [39] with the following amplification conditions: an initial denaturation cycle at 94 °C (5 min), 30 denaturation cycles at 94 °C (1 min), an annealing cycle at 56 °C (30 s), an extension cycle at 72 °C (2 min), and a final extension cycle at 72 °C (10 min). In order to evaluate the quality of the reaction, a 1% agarose gel electrophoresis was performed, then visualized using a blue light transilluminator and sent to Macrogen Inc. (Seoul, Republic of Korea) for sequencing. Electropherograms were reviewed and edited using the software CLUSTALX 2.0 [40] and SEAVIEW [41]. The resulting sequences were compared in the GenBank database using the BLAST program accessed on 15 July 2022 (http://blast.ncbi.nlm.nih.gov/Blast.cgi). The sequences of the reference strains were obtained from the EzBioCloud platform [42] to complete the phylogenetic analysis. A multiple alignment was performed using CLUSTALX 2.0 and the acquired sequences were edited using SEAVIEW. The phylogenetic analysis was performed using the neighbor-joining (NJ) method included in the program MEGA 11 [43].

### 3.5. Evaluation of Tolerance to Salinity, pH and Sodium Capture Capacity

The isolates were grown at 0, 2.5, 5, 7.5, 10, 15, 20 and 25% of NaCl by streaking in nutrient agar medium and were incubated at 28 °C ± 2 °C for seven days [44,45], after which growth was measured as excellent (+++, for diameter > 5 mm), moderate (++, for 2 mm < diameter ≤ 5 mm), little (+, for diameter ≤ 2 mm) and no growth (−); this analysis was performed in triplicate. To determine the optimal pH for the growth of the isolates, their development was evaluated at pH levels of 5, 6, 7, 8, 9, 10 and 11 [44,45], determining growth according to the abovementioned criteria (excellent, moderate, little, no growth), with the tests performed in triplicate. The sodium capture capacity of the bacteria was determined using the method described by Damodaran et al. [46], cultivating the bacteria in LB (Luria–Bertani) medium with a NaCl concentration of 400 mM at 30 °C for 24 h. Subsequently, the bacterial cells were centrifuged at 12,000 rpm, the supernatant was removed and the sodium absorption was measured by flame photometry using the atomic absorption spectrometer GBC SensAA. This analysis was performed in triplicate.

### 3.6. Production of Characteristic Growth Promotion Metabolites and Enzymatic Activity of the Isolates

The phosphate solubilization capacity (PO_4_^3−^) was determined in triplicate following the method described by Rafi et al. [47], using SRS medium (C_6_H_12_O_6_ 10 g/L; KCl 0.2 g/L; (NH_4_)SO_4_ 0.5 g/L; NaCl 0.2 g/L; MgSO_4_X7H_2_O 0.3 g/L; FeSO_4_X7H_2_O 0.002 g/L; MnSO_4_ 0.004 g/L; yeast extract 0.5 g/L), modified with Ca_3_(PO_4_)_2_ 2 g/L. After 48 h of incubation at 30 °C, the concentration of available phosphorus was measured using the multiparameter photometer HANNA HI-83200 at 420 nm.

IAA production was determined by Salkowski’s colorimetric reaction [48], inoculating the strains in tryptic soy broth (TSB) medium (C_6_H_12_O_6_ 5 g/L; K_2_HPO_4_ 1 g/L; (NH_4_) NO_3_ 0.4 g/L; NaCl 0.2 g/L; MgSO_4_X7H_2_O 0.4 g/L; Tryptone 20 g/L) supplemented with 1% l-tryptophan (*w/v*) and without l-tryptophan. The strains were incubated at 30°C for 48 h; after which IAA was quantified in a BioTek plate reader at 530 nm. This analysis was performed in triplicate.

The production of NH_4_^+^ from organic sources was determined, in triplicate, following the methodology of Cappuccino and Welsh [49], and was quantified using the TNT 830 kit and the multiparameter photometer HANNA HI-83200 at 655 nm. 

The carbonate solubilization capacity was determined in a modified Foster’s medium with CaCO_3_ at a concentration of 1.5 g/L, where the presence of a halo was considered as positive. This analysis was performed in triplicate. For the solubilization of zinc, NBRIP medium was used [50], modifying the Ca_3_(PO_4_)_2_ by ZnO at a concentration of 2 g/L, where the formation of a translucent halo around the bacterial colony indicates a positive result. This analysis was performed in triplicate. In addition, the activity coefficient (Wact) of zinc and carbonate solubilization was determined using the equation proposed by Hrynkiewicz et al. [51] as follows:

Wact = Sh2/(Sc × t)

where Sh is the diameter of the hydrolysis zone, Sc is the diameter of the bacterial colony and t is the incubation time (days).

Proteolytic enzyme activity was determined using LB medium supplemented with 2% skim milk powder, where the formation of translucent halos around the colonies after 72 h of incubation at 30 °C indicated the production of proteases [52]. The lipolytic activity was determined in plates of LB medium supplemented with 1% olive oil, previously emulsified by ultrashaking for 15 min. The plates were incubated at 30 °C for five days, after which the formation of translucent halos was considered positive for lipases. These analyzes were performed in triplicate. 

Cellulolytic activity was determined by seeding the bacterial isolates in nutrient agar supplemented with 0.5% carboxymethyl cellulose. The plates were incubated at 30 °C for seven days; subsequently, they were covered with Lugol’s iodine as a revealer, and after 15 min, the cultures were checked for formation of clear halos around the colonies which indicate a positive result for cellulases [50]. The production of amylases was determined in LB medium plates supplemented with 0.2% modified starch. The plates were incubated at 30 °C for seven days, after which they were covered with Lugol’s iodine solution, with the production of clear zones around the colonies indicating a positive result for amylase production [53]. These analyzes were performed in triplicate.

### 3.7. Promotion of Plant Growth in Germination of Solanum lycopersicum under Saline Stress

The bacteria evaluated in this trial were selected based from those that presented at least five growth promotion characteristics and had better results in solubilization of phosphorus, AIA and AIAt. For this test, the bacteria *Halomonas* sp. SVCN6, *Bacillus* sp. SVHM1.1 and *Halomonas* sp. SVHM8 were selected for evaluation. These were grown in nutrient broth at 28 °C and adjusted to an optical density of 1.0 at 625 nm for subsequent application to seeds.

For this study, *Solanum lycopersicum* seeds were used as a model plant. The seeds were sterilized with 70% ethanol for 1 min. The ethanol was then removed and the seeds were disinfected with 1% sodium hypochlorite and stirring at 130 rpm for 10 min; finally, washings were performed to remove the excess hypochlorite [25]. The seeds were inoculated with each of the bacteria chosen for this analysis as follows. Firstly, the seeds were added to 10 milliliters of the inoculum previously adjusted in absorbance and shaken for 60 min at 130 rpm and then dried at room temperature. The seeds were placed in a petri dish containing water agar medium at different concentrations of salinity (0, 20, 60 and 100 mM NaCl) and with the following treatments: control, *Halomonas* sp. SVCN6, *Bacillus* sp. SVHM1.1 y *Halomonas* sp. SVHM8; 25 seeds were incubated per treatment at 25°C with 16 h of light/8 h of darkness in a germination chamber. The experiment was carried out in triplicate. It was monitored daily until reaching a germination percentage that did not vary from day to day (for this trial it was 12 days). To evaluate the efficiency of the treatments, the germination percentage was determined in each treatment [54], and the results were compared for each salinity level.

### 3.8. Statistical Analysis

For the growth promotion characteristics shown by the bacteria and sodium capture, the results shown are expressed as mean values ± standard deviation of the error withanalyzes performed in triplicate. The germination evaluation was performed in triplicate and analyzed by means of one-way ANOVA and Tukey’s test (*p* < 0.05) to determine the significant mean differences between the bacteria evaluated, using the MINITAB statistical software (version 17 for Windows).

## 4. Discussion

According to the United States Salinity Laboratory, saline-sodic soils have an electrical conductivity above 4 dS/m and an ESP above 15%, as well as high concentrations of sulfates and chlorides [35]. The soil analyzed in this study presented values that match this description, with a pH of 8.5, electrical conductivity of 23.5 dS/m, ESP of 40.6 and SAR of 47.3 dS/m; such characteristics compromise plant nutrition and growth [35,55]. High levels of salinity in the soil are a determining factor in the recruitment of halotolerant microorganisms that in association with the rhizosphere of halophytic plants can increase tolerance to salt stress [18,25]. Thanks to various biochemical and physiological mechanisms, the halophyte *S. verrucosum* has excellent growth in soils affected by salinity. Its adaptive capacity to resist this type of abiotic stress is attributable to its association with soil bacteria that have the capacity to promote plant growth [10]. However, there are few studies describing the microorganisms that are naturally associated with the rhizosphere of *S. verrucosum* and that could directly or indirectly generate mechanisms for increasing tolerance to salt stress.

In the analyzed sequences of bacteria isolated from the rhizosphere of *S. verrucosum*, the predominant families were Firmicutes, Proteobacteria and—to a lesser extent—Actinobacteria, while the predominant genera were *Halomonas* and *Bacillus*. These findings agree with other works which report that the bacterial community of the rhizosphere of halophytic plants consists of bacteria belonging the phyla Proteobacteria, Actinobacteria [18,24,56] and Firmicutes [57]. The percentages of the prevailing phyla depend on the salinity concentration of the soil to which the plant is subjected [58]. Szymanska et al. [25] investigated the diversity of phyla to which the rhizospheric or endophytic bacteria of the halophyte *Aster Tripolium* belong, and found that the abovementioned phyla were the most representative, along with Bacteroidetes. Yamamoto et al. [59] isolated and characterized bacteria from the rhizosphere and root endosphere of the halophytes *Glaux maritima* and *Salicornia europaea* and reported that Proteobacteria, Actinobacteria and Bacteroidetes were the predominant phyla. Fidalgo et al. [57] identified that the dominant phyla of the bacteria associated with *Halimione portulacoides* were Proteobacteria and Actinobacteria; additionally, Firmicutes represented 10% of the bacterial isolates. This agrees with the results of the present study, which showed that these three phyla are representative of the bacterial community of the rhizosphere of *S. verrucosum*. The genera of bacteria are also dependent on the degree of salinity; for example, a study showed that the predominant genera in the rhizosphere of *Salicornia europaea* were *Bacillus* sp., *Streptomyces* sp., *Microbacterium* sp., *Serratia* sp. and *Salinicola* sp. [18]. Sanjay et al. [6] noted that the prevailing genera of endophytic bacteria of two halophytic plant species (*Sphaeranthus indicus* and *Salicornia brachiata*) were *Bacillus* sp., whereas Hrynkiewicz et al. concluded that *Pseudomonas* and *Serratia* spp. were the most prevalent bacterial genera associated with *Salix viminalis* [51]. In another work related to the rhizobacteria of halophytes, a higher percentage of genera belonging to *Bacillus* was found [15]. Moreover, the genus *Halomonas* has also been identified as representative of bacteria associated with halophytic plants and saline soils [60,61].

Rhizospheric bacteria that exhibit growth-promoting characteristics are bacteria that inhabit the rhizosphere of plants and facilitate the growth of their host by means of various direct and indirect mechanisms [2,12]. These mechanisms include the ability of these bacteria to fix nitrogen, solubilize phosphate as well as produce secondary metabolites (such as exopolysaccharides and osmolytes), phytohormones, enzymes and siderophores [12,62] that regulate the plant’s defense and activate antioxidant enzymes under salt stress [2,62]. In this study, bacteria associated with the root soil of *S. verrucosum* were characterized, showing positive results for tolerance to different concentrations of NaCl, growth capacity at different pH levels, plant growth promoting characteristics such as IAA production, phosphorus and carbonate solubilization, NH_4_^+^ production from organic sources and Na^+^ capture capacity, as well as enzymatic activity of proteases, lipases, amylases and cellulases.

Most of the isolates from the rhizosphere of *S. verrucosum* presented growth capacity at NaCl concentrations between 0% and 15%, and at pH levels between 6 to 11. Of the isolated bacteria, 15% were halophilic and 85% were halotolerant. In order to grow, halophilic bacteria need NaCl in the medium, whereas halotolerant bacteria can grow in medium with 0 to 25% NaCl [12]. The halotolerant bacteria associated with the rhizosphere of halophytes have a great capacity to survive in high concentrations of salinity [18,21,63]. Wu et al. [20] isolated *Bacillus safensis* from the rhizosphere of *Chloris virgata*, grew it at different concentrations of NaCl and observed growth capacity up to a concentration of 12% NaCl. El-Awady et al. [10] isolated bacteria from the rhizosphere of *S. verrucosum*, confirming tolerance to NaCl concentrations ranging from 0.5 to 10%. The present study identified bacteria that grew at concentrations above 10%, tolerating up to 20% NaCl (*Marinococcus* sp. isolate SVHM5). Besides being able to survive in high concentrations of salinity, halophilic bacteria showed growth at different pH levels, ranging from 4 to 12 [21,60]. These types of stress (NaCl and pH) can directly or indirectly compromise the growth of plants. Fortunately, bacteria can protect host plants due to their capacity to tolerate salt and pH stress [21].

Singh and Jha [23] isolated *Serratia marcescens* from the rhizosphere of *Capparis decidua*, demonstrating its capacity to solubilize phosphates and produce IAA, siderophores and ammonium. Roman-Ponce et al. [50] obtained bacteria from the rhizosphere of two species of plants that grow under conditions of metal contamination, showing the growth promoting potential of 76% of the isolates, which had the capacity to solubilize phosphate, and produce siderophores and IAA. Previous works have also shown that there are bacteria in the rhizosphere of halophytic plants that have plant growth-promoting characteristics [6,14,15]. The Salkowki colorimetric test for the determination of IAA production by plant growth-promoting bacteria is one of the most used due to its simplicity and economy, but in addition to being reactive for IAA by the tryptophan-dependent pathway, it is also indicative of other indolic compounds such as AIP and AIM [32], with the AIP and AIM pathway being the main routes for the synthesis of AIA by bacteria since the main synthesis pathways culminate in the production of AIA [33]. Likewise, the present study evidenced the capacity of bacteria to solubilize zinc, an element of great agricultural importance since it is essential for the activity of many enzymes. Zinc in saline soils is a limited element; therefore, the use of solubilizing bacteria could become a great resource to improve plant nutrition [64]. An alternative for the recovery of saline soils is the release of calcium from native calcium carbonate which—by exchange with sodium in the soil—can reduce the sodium concentration by subsequent washing [65]. These bacterial plant growth-promoting mechanisms exist due to the production of organic substances that are capable of causing responses in plant cells at biochemical, physiological and morphological levels [66], which in addition to participating in biogeochemical cycles, make organic and inorganic compounds available that can be later used by plants subjected to salt stress [60].

Furthermore, the sodium capture capacity of bacteria from the root soil of *S. verrucosum* has not been reported in any previous work. Sánchez-Leal and Argullo-Arias [67], using the atomic absorption spectroscopy technique, found four halophilic bacteria that had the ability to capture sodium in vitro, proposing these bacteria as alternatives for the bioremediation of saline soils. In another study, bacteria with the capacity to capture sodium were isolated from rhizospheric soil samples of rice plants grown in areas affected by high salinity levels, and *Bacillus tequilensis* in particular presented the highest sodium capture when subjected to different NaCl concentrations ranging from 0 to 2 M [63]. The capacity of halotolerant and halophilic bacteria to accumulate ions such as Na^+^ is attributable to their need to balance the osmotic pressure of the medium by developing specific proteins that are stable and active in the presence of salts. Likewise, moderate halophilic bacteria accumulate high concentrations of osmolytes, providing osmotic balance [68].

Furthermore, 60% of the bacteria in this study presented some enzymatic activity (lipases, proteases, amylases and/or cellulases). This agrees with the findings of many authors who have reported the enzymatic activity of halophilic and halotolerant bacteria of the genera *Bacillus* [13,15], *Halobacillus* [15], *Vigibacillus* [60], *Oceanobacillus* [15,60]. These are excellent producers of enzymes, especially lipases, amylases, proteases, cellulases, gelatinases and catalases [60], or have antioxidant enzyme activity [6,11,17]. The enzymes produced by bacteria play an important role in the hydrolysis of high molecular weight biopolymers, which are the beginning of the metabolism of organic compounds in the different ecosystems due to their relevance in the biogeochemical cycles [68]. Considering that all the biochemical reactions in the soil are catalyzed by enzymes, it can be stated that a high enzymatic activity is indicative of soil biological activity [69]. Chaudhary et al. [19] isolated rhizobacteria that presented enzymatic activity (cellulases, b-glucosidases and ureases) which can serve as an early indicator of changes in soil fertility due to their relationship with carbon and nitrogen mineralization. The high activity of certain enzymes could induce high decomposition of organic material, resulting in greater availability of nutrients in the rhizosphere [70], which would be used by the halophyte and help it improve its resistance to salt stress. Mukhtar et al. [60] reported the hydrolytic enzyme activity (amylases, proteases, lipases and cellulases) of halophilic bacteria as an alternative source of enzyme production in the pharmaceutical, food and paper industry [71].

The inoculation of bacteria in *Solanum lycopersicum* seeds under salt stress had a greater influence at 100 mM (salinity evaluated at higher concentration), and similar results have been reported in other studies [27,50]. Kearl et al. [22] inoculated bacteria isolated from halophytic plants to alfalfa seeds, observing an increase in growth compared to non-inoculated seeds in the presence of salinity. Sharma et al. [72] evaluated bacteria from the halophyte *Arthrocneum indicum* in peanut germination under saline stress and observed improved growth with respect to non-inoculated controls. The influence of bacteria with plant growth promotion characteristics on plant growth under stressful conditions is attributed to several mechanisms such as the ability to produce ammonium, phosphorus solubilization, and the production of osmolytes and phytohormones, which provide the host plant the adaptive capacity of resistance to salt stress [2,10,11,12,62]. In addition, the synthesis of IAA is one of the mechanisms with the greatest influence because this hormone is responsible for regulating aspects of plant growth such as root initiation, cell elongation, and increase in the root surface for nutrient uptake [72], characteristics that improve germination under this type of stress.

## 5. Conclusions

In this study, bacteria from the root soil of *Sesuvium verrucosum* Raf. were isolated and characterized. The predominant phyla identified were Proteobacteria, Firmicutes and Actinobacteria. It was determined that 85% of the root soil isolates were halotolerant bacteria and 15% were halophilic bacteria, which showed growth at different pH levels and NaCl concentrations, some surviving at concentrations higher than 20%. In addition, different morphological characteristics were observed among the bacteria. Regarding the growth promotion traits, the majority of bacteria showed phosphorus solubilization capacity and nitrogen production from an organic source; 50% of the bacteria had the ability to produce IAA by the tryptophan-dependent (AIAt) and tryptophan-independent (IAA) pathways. The capacity for carbonate solubilization was also observed. These characteristics were evident in the germination tests where *Halomonas* sp. SVCN6, *Bacillus* sp. SVHM1.1 and *Halomonas* sp. SVHM8 showed a positive influence on germination percentage at the highest level of salinity evaluated.

## Figures and Tables

**Table 1:** Salinity characterization of the root soil of *S. verrucosum* measured in a saturated paste extract.

Parameters	Concentration
Electrical conductivity (dS/m)	23.5 ± 0.65
pH	8.5 ± 0.2
Ca^2+^ (meq/L)	24.6 ± 2.8
Mg^2+^ (meq/L)	13.4 ± 2.0
K^+^ (meq/L)	9.93 ± 0.5
Na^+^ (meq/L)	206 ± 5.5
SO_4_^−^ (meq/L)	111 ± 3.1
Cl^−^ (meq/L)	138 ± 2.7
HCO_3_^−^ (meq/L)	3.87 ± 0.4
CO_3_^−^ (meq/L)	2.71 ± 0.2
SAR	47.3 ± 1.8
ESP	40.6 ± 1.0

The parameters were determined based on the saturated paste extract. dS/m = deciSiemens per meter; meq/L = milliequivalents per liter; SAR = sodium adsorption ratio; ESP = exchangeable sodium percentage. Values indicate the mean ± standard deviation of the three replicates.

**Table 2:** Growth of the isolates at different pH levels.

ISOLATE	pH						
	5	6	7	8	9	10	11
*Bacillus* sp. SVCN1	-	++	++	+++	++	-	-
*Bacillus* sp. SVHM1.1	-	++	++	+++	+++	++	++
*Bacillus* sp. SVHM9	+	+++	+++	+++	+++	++	-
*Bacillus subtilis* SVCN10	-	+	++	+++	+	+	+
*Bacillus subtilis* SVHM10	+	+++	+++	+++	+++	++	+
*Oceanobacillus* sp. SVCN2	+	+	++	++	+++	++	++
*Oceanobacillus* sp. SVHM7	-	+	++	+	+	+	+
*Staphylococcus epidermidis* SVHM1	-	+	+++	++	+	-	-
*Marinococcus* sp SVHM5	+	+++	+++	+++	+++	+++	++
*Nocardiopsis* sp SVHM6.2	-	-	++	-	-	-	-
*Halomonas* sp. SVCN3	-	+	+++	++	++	++	++
*Halomonas* sp. SVCN4	-	++	++	++	+++	++	++
*Halomonas* sp. SVCN6	+	++	++	+++	++	++	++
*Halomonas* sp. SVCN8	+	++	++	+++	++	++	++
*Halomonas* sp. SVHM2	-	++	++	+++	++	++	++
*Halomonas* sp. SVHM3	+	++	++	+++	++	++	++
*Halomonas* sp. SVHM4	+	++	++	+++	+++	++	++
*Halomonas* sp. SVHM8	-	++	++	++	+++	++	++
*Halomonas huangheensis*SVCN7	-	++	++	+++	+++	++	++
*Halomonas huangheensis*SVHM6	-	+	++	+	-	-	-

Symbols: (-) No growth; (+) Little growth; (++) Moderate growth; (+++) Excellent growth.

**Table 3:** Growth at different concentrations of NaCl.

ISOLATE	%NaCl—(mol/L)							
	0	2.5 (0.42)	5 (0.85)	7.5 (1.28)	10 (1.71)	15 (2.56)	20 (3.42)	25 (4.27)
*Bacillus* sp. SVCN1	++	++	+++	+++	+++	-	-	-
*Bacillus* sp. SVHM1.1	++	++	++	+++	+++	+	-	-
*Bacillus* sp. SVHM9	+++	+++	+++	+++	++	+	-	-
*Bacillus subtilis* SVCN10	+++	++	++	++	++	-	-	-
*Bacillus subtilis* SVHM10	+++	+++	+++	+++	++	-	-	-
*Oceanobacillus* sp. SVCN2	-	++	++	+++	+++	++	-	-
*Oceanobacillus* sp. SVHM7	+	+	+	++	++	-	-	-
*Staphylococcus epidermidis* SVHM1	+++	+++	++	++	++	-	-	-
*Marinococcus* sp. SVHM5	++	++	++	+++	+++	+++	++	+
*Nocardiopsis* sp. SVHM6.2	-	-	+	+	++	-	-	-
*Halomonas* sp. SVCN3	-	++	++	+++	+++	++	-	-
*Halomonas* sp. SVCN4	++	++	++	+++	+++	+++	+	-
*Halomonas* sp. SVCN6	++	++	+++	+++	+++	++	+	-
*Halomonas* sp. SVCN8	++	++	++	+++	+++	+++	-	-
*Halomonas* sp. SVHM2	++	++	+++	+++	+++	+++	++	-
*Halomonas* sp. SVHM3	++	++	++	+++	+++	++	+	-
*Halomonas* sp. SVHM4	++	+++	+++	+++	+++	++	+	-
*Halomonas* sp. SVHM8	++	++	+++	++	++	++	++	-
*Halomonas huangheensis*SVCN7	++	++	+++	+++	+++	++	+	-
*Halomonas huangheensis*SVHM6	++	++	++	+++	++	-	-	-

Symbols: (-) No growth; (+) Little growth; (++) Moderate growth; (+++) Excellent growth.

## Data Availability

Not applicable.

## Supplementary Materials

The following supporting information can be downloaded at: https://www.mdpi.com/article/10.3390/plants11233355/s1, Table S1: Morphological and microscopic characteristics of the isolates; Table S2: Identification of bacterial isolates from the root soil of *Sesuvium verrucosum*; Table S3. Plant growth promotion capacity of bacteria from the root soil of *Sesuvium verrucosum*.
