# Supplementary material for: Identification of Halophilic and Halotolerant Bacteria from the Root Soil of the Halophyte Sesuvium verrucosum Raf
Source: Plants (Basel). 2022 Dec 2;11(23):3355. doi: 10.3390/plants11233355 (PMC9740589; doi:10.3390/plants11233355)
Supplement: Supplementary file 1 [file plants-11-03355-s001.zip › plants-1976680-supplementary.pdf]

Table S1. Morphological and microscopic characteristics of the isolates.

ISOLATE	SIZE (mm)	FORM	ELEVATION	MARGIN	SURFACE	COLOR	CONSISTENCY	STAINING REACTION	SHAPE	ARRANGEMENT
SVCN1	3 - 6	Oval	Flat	Entire	Smooth	Cream	Creamy	Gram-positive	Bacillus	Streptobacillus
SVCN2	1 - 2	Irregular	Raised	Undulate	Rough	Orange	Creamy	Gram-positive	Coccus	Staphylococcus
SVCN3	1 - 2	Circular	Raised	Entire	Smooth	Cream	Creamy	Gram-negative	Bacillus	Bacillus
SVCN4	1 - 6	Irregular	Flat	Entire	Smooth	Cream	Creamy	Gram-negative	Coccus	Bacillus
SVCN6	≤ 1	Irregular	Flat	Lobate	Smooth	Cream	Creamy	Gram-positive	Bacillus	Bacillus
SVCN7	1 - 2	Irregular	Raised	Undulate	Smooth	Cream	Creamy	Gram-negative	Bacillus	Diplobacillus
SVCN8	2 - 4	Irregular	Flat	Lobate	Rough	Cream	Dry	Gram-negative	Bacillus	Bacillus
SVCN10	2 - 6	Irregular	Flat	Undulate	Smooth	Cream	Dry	Gram-negative	Bacillus	Diplobacillus
SVHM1	4 - 7	Circular	Raised	Entire	Smooth	Yellow	Creamy	Gram-positive	Bacillus	Bacillus
SVHM1.1	2 - 6	Circular	Flat	Undulate	Rough	Yellow	Dry	Gram-positive	Bacillus	Bacillus
SVHM2	2 - 3	Irregular	Flat	Undulate	Rough	Yellow	Creamy	Gram-negative	Bacillus	Bacillus
SVHM3	≤ 3	Irregular	Flat	Undulate	Smooth	Cream	Creamy	Gram-negative	Bacillus	Diplobacillus
SVHM4	≤ 4	Irregular	Raised	Entire	Rough	Cream	Creamy	Gram-negative	Coccus	Staphylococcus
SVHM5	1 - 2	Circular	Convex	Entire	Smooth	Orange	Creamy	Gram-positive	Coccus	Staphylococcus
SVHM6	5 - 10	Punctiform	Flat	Entire	Rough	Cream	Dry	Gram-positive	Bacillus	Bacillus
SVHM6.2	2 - 6	Circular	Flat	Entire	Smooth	Cream	Dry	Gram-negative	Bacillus	Bacillus
SVHM7	≤ 1	Punctiform	Convex	Entire	Smooth	Cream	Creamy	Gram-positive	Coccus	Staphylococcus
SVHM8	3 - 6	Irregular	Flat	Entire	Smooth	Yellow	Creamy	Gram-negative	Bacillus	Bacillus
SVHM9	4 - 7	Irregular	Flat	Entire	Rough	Cream	Dry	Gram-negative	Bacillus	Palisade
SVHM10	4 - 7	Irregular	Flat	Entire	Rough	Cream	Dry	Gram-negative	Bacillus	Palisade

Table S2. Identification of bacterial isolates from the root soil of *Sesuvium verrucosum*

**Isolate (NCBI accession number)**		**Related species and accession number**	**% of Identity**
SVCN1	*Bacillus* sp. (ON571718)	*Bacillus cabrialesii* Si428 (OM866230.1)	99.93%
		Bacillus sp. TZ29 (KR265733.1)	99.93%
SVCN2	*Oceanobacillus* sp. (ON571719)	*Halophilic bacterium* QW107 (EU124355.1)	99.79%
		Oceanobacillus sp. KGS-44-15 (KU840475.1)	99.79%
SVCN3	*Halomonas* sp. (ON571720)	*Halomonas heilongjiangensis* 1078 (MN712572.1)	99.46%
		Halomonas salina T8 (KX389562.1)	99.43%
SVCN4	*Halomonas* sp. (ON571721)	*Halomonas* sp. NIMD7 (LC189163.1)	99.43%
		Halomonas boliviensis kknpp_38 (MK100425.2)	99.35%
SVCN6	*Halomonas* sp. (ON571722)	*Halomonas* sp. 8CN1-1 (MW757276.1)	99.12%
SVCN7	*Halomonas huangheensis* (ON571723)	*Halomonas huangheensis* AS4H13 (KP706816.1)	99.71%
SVCN8	*Halomonas* sp. (ON571724)	*Halomonas sulfidaeris* W13 (MN746145.1)	99.26%
		Halomonas sp. KYW1415 (MH782071.1)	99.19%
SVCN10	*Bacillus subtilis* (ON571725)	*Bacillus subtilis* At3 (CP051462.1)	99.93%
		Bacillus subtilis subsp. inaquosorum T1 (MT339257.1)	99.93%
SVHM1	*Staphylococcus epidermidis* (ON571726)	*Staphylococcus epidermidis* 3039 (MT613456.1)	99.85%
SVHM 1.1	*Bacillus* sp. (ON571727)	*Bacillus filamentosus* Y16 (MK721036.1)	99.93%
		Bacillus sp. L37 (MG594627.1)	99.86%
SVHM2	*Halomonas* sp. (ON571728)	*Halomonas* sp. NIMD18 (LC189171.1)	99.28%
SVHM3	*Halomonas* sp. (ON571729)	*Halomonas cupida* LM4H63 (KP706813.1)	99.78%
		Halomonas sp. KKDK-1 (KF682368.1)	99.64%
SVHM4	*Halomonas* sp. (ON571730)	*Halomonas* sp. NIMD7 (LC189163.1)	99.07%

SVHM5	*Marinococcus* sp. (ON571731)	*Marinococcus* sp. (AY987847.1)	99.44%
		Marinococcus halophilus JCM 2473 (LC379135.1)	99.31%
SVHM6	*Halomonas huangheensis* (ON571732)	*Halomonas huangheensis* AS4H13 (KP706816.1)	99.93%
SVHM6.2	*Nocardiopsis* sp. (ON571733)	*Nocardiopsis chromatos* LK7 (MT669277.1)	98.94%
		Nocardiopsis chromatos DYB142 (MK968676.1)	98.89%
SVHM7	*Oceanobacillus* sp. (ON571734)	*Oceanobacillus sp.* OS11 (MZ818023.1)	99.86%
		Oceanobacillus picturae SQA-14 (MT110647.1)	99.86%
SVHM8	*Halomonas* sp. (ON571735)	*Halomonas* sp. P56 (EU880513.1)	99.49%
SVHM9	*Bacillus* sp. (ON571736)	*Bacillus subtilis subsp. spizizenii* NM163 (MT114506.1)	99.09%
		Bacillus subtilis SCAU-Z8 (MW346180.1)	99.09%
SVHM10	*Bacillus subtilis* (ON571737)	*Bacillus subtilis* HDXJ04 (KT201603.1)	97.72%

Table S3. Plant growth promotion capacity of bacteria from the root soil of *Sesuvium verrucosum*.

ISOLATE	Solub. PO_4^{3-} (mg/l)	Prod. IAA (µg/ml)	Prod. IAAt (µg/ml)	Prod. NH_4^+ (mg/ml)	Solub. CO_3^- (*Wact*)	Solub. Zn^+ (*Wact*)	Capture of Na^+ (mEq)	Lipases	Proteases	Amylases	Cellulases
Bacillus sp. SVCN1	21 ± 3.1	-	-	21.5 ± 1.0	3.5 ± 0.6	-	24 ± 8	-	-	+	+
Bacillus sp. SVHM1.1	81 ± 4.4	7.7 ± 2.3	17.6 ± 1.8	10.3 ± 0.4	-	0.7 ± 0.1	23 ± 4	-	-	-	-
Bacillus sp. SVHM9	12 ± 1.5	-	-	9.4 ± 1.4	-	-	15 ± 4	-	-	+	+
Bacillus subtilis SVCN10	46 ± 3.1	-	-	17.8 ± 1.5	8.5 ± 2.1	-	11 ± 3	-	-	+	+
Bacillus subtilis SVHM10	81 ± 1.0	-	-	19.1 ± 1.2	-	-	11 ± 2	-	-	+	+
Oceanobacillus sp. SVCN2	23 ± 0.6	-	-	-	-	-	10 ± 1	+	-	-	-
Oceanobacillus sp. SVHM7	61 ± 7.2	-	-	-	-	-	13 ± 3	-	-	-	-
Staphylococcus epidermidis SVHM1	49 ± 2.5	-	4.9 ± 0.4	15.4 ± 0.8	-	-	19 ± 5	-	-	-	-
Marinococcus sp. SVHM5	34 ± 6.1	-	-	-	2.9 ± 0.6	-	36 ± 8	+	-	-	-
Nocardiopsis sp. SVHM6.2	30 ± 2.3	-	-	-	-	-	19 ± 3	-	-	-	-
Halomonas sp. SVCN3	12 ± 3.2	9.9 ± 1.5	16.1 ± 5.4	5.5 ± 2.5	1.8 ± 0.4	-	26 ± 8	-	-	-	+
Halomonas sp. SVCN4	11 ± 2.1	7.7 ± 1.2	15.4 ± 2.1	14.6 ± 0.6	2.7 ± 0.3	-	-	-	+	-	-
Halomonas sp. SVCN6	46 ± 7.4	6.6 ± 0.9	14.3 ± 1.7	16.4 ± 0.3	1.5 ± 0.4	-	-	-	+	-	-
Halomonas sp. SVCN8	-	-	-	13.5 ± 2.2	2.0 ± 0.2	-	17 ± 8	+	-	-	-
Halomonas sp. SVHM2	30 ± 1.7	6.8 ± 0.5	18.6 ± 4.4	14.4 ± 0.3	6.2 ± 1.8	-	-	+	-	-	-
Halomonas sp. SVHM3	9 ± 2.6	4.0 ± 1.8	16.6 ± 0.8	-	-	-	12 ± 2	-	-	-	-
Halomonas sp. SVHM4	69 ± 2.0	7.3 ± 0.9	13.8 ± 3.4	17.8 ± 1.5	-	-	-	+	-	-	-
Halomonas sp. SVHM8	50 ± 3.1	8.9 ± 1.2	20.6 ± 3.8	17.8 ± 0.5	6.7 ± 0.4	-	34 ± 2	-	-	-	-
Halomonas huangheensis SVCN7	35 ± 1.5	6.1 ± 1.9	15.6 ± 2.7	-	1.7 ± 0.1	-	23 ± 3	+	-	+	-
Halomonas huangheensis SVHM6	61 ± 2.2	-	-	11.7 ± 0.5	-	-	38 ± 2	-	-	-	-

Phosphate solubilization (Solub. PO_4^{3-}), Tryptophan-independent IAA production (Prod. IAA), Tryptophan-dependent IAA production (Prod. IAAt), Ammonium production from organic sources (Prod. NH_4^+), Carbonate solubilization (Solub. CO_3^-), Zinc solubilization (Solub. Zn^+), Sodium capture capacity (Capture of Na^+). Signs: positive capacity (+), negative capacity (-). Values indicate the mean ± standard deviation of the three replicates.
